# Successful flattening of COVID-19 epidemiological curve in Jordan

**DOI:** 10.7189/jogh.10.020361

**Published:** 2020-12

**Authors:** Mohammad Al-Tamimi, Rami Qaisieh, Ramzy Edward Tadros, Marwan Shalabi, Anas Alhasoun, Muna M Kilani

**Affiliations:** 1 Department of Basic Medical Sciences, Faculty of Medicine, Hashemite University, Zarqa, Jordan; 2 Department of General and Specialized Surgery, Faculty of Medicine, Hashemite University, Zarqa, Jordan; 3 Department of General and Specialized Surgery, Prince Hamzah Hospital, Ministry of Health, Amman, Jordan; 4 Department of Pediatrics and Neonatology, Faculty of Medicine, Hashemite University, Zarqa, Jordan; 5 Department of Pediatrics and Neonatology, Prince Hamzah Hospital, Ministry of Health, Amman, Jordan

Humans are currently experiencing the catastrophic effects of the Coronavirus disease 2019 (COVID-19) pandemic. As of 16 April 2020, the disease has spread in more than 200 countries with over 2 million confirmed cases, over 140 000 deaths, and over 0.5 million recovered cases [1,2]. USA, Spain and Italy are currently registering the highest number of confirmed cases [1,2]. The Eastern Mediterranean Region are currently experiencing the surge of the disease with over 110 000 confirmed cases and Iran being strongly affected with over 75 000 confirmed cases [1,2]. The epidemiological features of COVID-19 disease vary significantly among different countries, populations, and timeline [1,3]. Furthermore, different control measures to combat COVID-19 infection have been applied by different authorities with variable success rates [3].

The COVID-19 incubation period ranges from 1 to 14 days [3,4]. The most common symptoms are fever, fatigue, dry cough, nasal congestion, sore throat, myalgia, and arthralgia [3,4]. Some patients may present with nausea, vomiting, and diarrhea [3,4]. Variations in frequency of symptoms, risk factors, and complications associated with COVID-19 have been reported by different studies [3,4]. Currently a real-time reverse transcriptase polymerase chain reaction (RT-PCR) test is widely used as the confirmatory test [3]. Lymphocytopenia, increased D-dimer level, prolonged prothrombin time (PT), elevated liver enzymes, creatinine, cardiac markers, C-reactive protein (CRP), interleukin-6, and erythrocyte sedimentation rate (ESR) have been reported with variabilities among COVID-19 patients [3,4].

## GLOBAL, REGIONAL, AND LOCAL COVID-19 EPIDEMIOLOGICAL SITUATION

As of 16 April 2020, the global total numbers of confirmed cases were 2 166 832, death cases were 144 515 (case-fatality rate 6.7%) and recovered cases were 546 269 (recovery rate 25.2%) [1,2] (**Table 1**). The highest number of total confirmed, recovered, and death cases was reported by the European Region at 1 011 369, 275 289, and 91 873, respectively, with the highest case-fatality rate (9.0%). While, the lowest number of total confirmed and death cases was reported by the Oceania Region at 7966, and 72 respectively, with the highest recovery rate (56.7%) and the lowest case-fatality rate (0.9%) [2] (**Table 1**). In the Eastern Mediterranean Region [1], the highest numbers of total confirmed, recovered, and death cases were registered in Iran at 77 995, 52 229, and 4869 respectively. While, the lowest number of total confirmed cases was reported by Sudan at 32 cases. The highest case-fatality rate was reported by Sudan and Algeria at 15.6% and 15.3% respectively, while the lowest case-fatality rate was reported in Qatar and Kuwait at 0.2%. The highest recovery rate was reported in Iran at 67.0%, while the lowest recovery rate was reported in Somalia at 2.5% [2] (**Table 1**).

**Table 1:** Global, regional and eastern Mediterranean Countries COVID-19 confirmed, recovered, and death cases with case-fatality rate and recovery rate (updated 16 April 2020)

Recovery rate	Total recovered cases	Case- fatality rate	Total death cases	Total confirmed cases	
25.2%	**546 269**	**6.7%**	**144 515**	**2 166 832**	**Global**
**Regions**					
**27.2%**	275** **289	9.0%	91** **873	1** **011** **369	Europe
**9.8%**	69** **977	5.1%	36** **191	716** **191	North America
**48.0%**	16** **451	3.6%	12** **389	344** **692	Asia
**38.5%**	25** **859	4.5%	3014	67** **091	South America
**24.1%**	4531	5.1%	962	18** **802	Africa
**56.7%**	4518	0.9%	72	7966	Oceania
**Eastern Mediterranean countries**					
**67.0%**	52** **229	6.2%	4,869	77** **995	Iran
**23.8%**	1645	1.8%	128	6919	Pakistan
**15.5%**	990	1.3%	83	6380	Saudi Arabia
**18.8%**	1095	0.6%	35	5825	United Arab Emirates
**10.1%**	415	0.2%	7	4103	Qatar
**22.3%**	596	7.3%	196	2673	Egypt
**10.9%**	249	5.7%	130	2283	Morocco
**34.5%**	783	15.3%	348	2268	Algeria
**41.4%**	703	0.4%	7	1700	Bahrain
**14.8%**	225	0.2%	3	1524	Kuwait
**59.7%**	856	5.6%	80	1434	Iraq
**17.3%**	176	0.4%	4	1019	Oman
**6.4%**	54	3.6%	30	840	Afghanistan
**5.2%**	43	4.5%	37	822	Tunisia
**8.1%**	54	3.2%	21	663	Lebanon
**12.4%**	73	0.3%	2	591	Djibouti
**64.4%**	259	1.7%	7	402	Jordan
**16.8%**	63	0.5%	2	374	Palestinian
**2.5%**	2	6.3%	5	80	Somalia
**15.2%**	5	6.1%	2	33	Syria
**22.9%**	11	2.1%	1	48	Libya
**12.5%**	4	15.6%	5	32	Sudan

## EPIDEMIOLOGICAL FEATURES OF COVID-19 IN JORDAN

### Timeline

The first case of confirmed COVID-19 in Jordan was registered on the 2nd of March 2020 for a Jordanian who had returned from Italy before quarantine procedures were applied. His symptoms resolved completely and had two negative COVID-19 PCR results before being discharged to home isolation on 14th March 2020 [1,5]. On 15th March, 11 new cases tested positive for COVID-19 including 5 Jordanians who had returned to the country, 6 French tourists, and one Iraqi. From that date, COVID-19 positive cases were registered daily at variable rates with the highest number registered on 26th March with 40 new cases and the lowest number of 0 cases registered on 10th April 2020 [5]. One more COVID-19 case recovered on the 26th March with a variable number of cases recovering daily afterward. The first death case due to COVID-19 was registered on 28th March (Table S1 in the **Online Supplementary Document**). The epidemiological status of COVID-19 was changed from imported cases to local transmission on 26th March 2020 [1].

**Figure:**
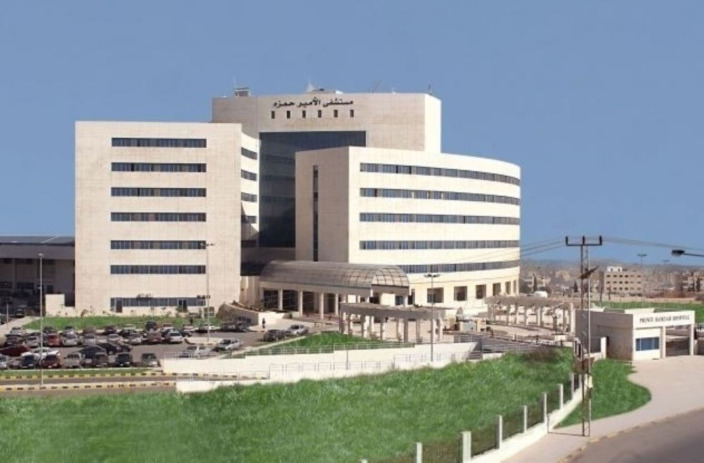
Photo: COVID-19 isolation center in Jordan – Prince Hamzah Hospital. Source: Ministry of Health Jordan.

### Current situation

As of 16th April 2020, the total numbers of confirmed, recovered, active, and death cases in Jordan were 402, 259 (64.4%), 136 (33.8%), and 7 (1.7%) respectively. The average number of daily new cases was 11.7 per day [5] (Table S1 in the **Online Supplementary Document**). The total number of COVID-19 tests administered was 20 500 RT-PCR tests (1.9 test per 1 million). Current COVID-19 incidence rate is 38.1 per 1 million (402 ∕ 10 554 000) and death rate is 0.66 per 1 million (7 ∕10 554 000) [5,6]. All the death cases were 60 years old or above and had documented chronic medical illnesses and die due to COVID-19 related complications including respiratory, cardiac and renal failure.

### Demographic analysis

Confirmed COVID-19 cases up to 31st March (n = 279) included 51% males, most cases were 20 to 59 years old (65%), Jordanians (88%), from the capital city of Amman (60.6%), and were admitted to PHH (57.3%) (Table S2 in the **Online Supplementary Document**). The largest identified COVID-19 cluster was from a wedding ceremony in Irbid who were in close contact with imported cases from Spain that resulted in 47 confirmed cases (17%) (Table S2 in the **Online Supplementary Document**). The highest number of cases were registered in Week 12 from the first case with 151 cases (55.1%) (Table S2 in the **Online Supplementary Document**). All cases had an incubation period of less than 14 days except for 3 cases (0.8%); 2 imported cases who were directly quarantined upon arrival to the airport and had symptoms at 17 and 18 days later and 1 local case of a medical staff who cared for a positive COVID-19 patient and had symptoms after 17 days of contact despite full quarantine for 2 weeks [5].

### Epidemiological curve

**Figure 1:**
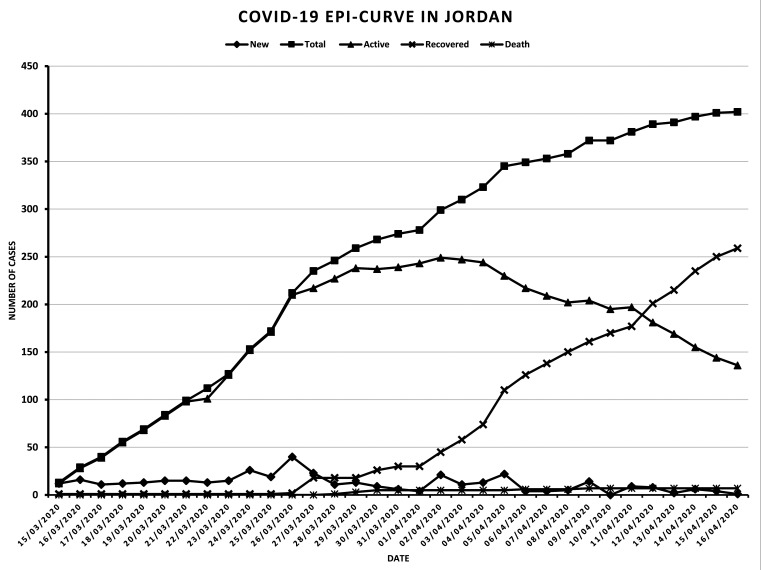
Epidemiological curve of daily COVID-19 cases in Jordan.

The epidemiological status of active COVID-19 cases for the last 4 weeks indicated a sharp increase from 15 to 26 March, followed by a marginal increase in number of active COVID-19 cases from 26 to 29 March, whilst the number of active cases became stable and reached a plateau from 29 March to 4 April, then active cases decreased until 16 April as the number of new cases decreased and is less than the number of recovered cases [5] (Table S1 in the **Online Supplementary Document****,** and **Figure 1**).

### Control measures

A large number of control measures were applied gradually from 27th February 2020 to mitigate and prevent the importation of COVID-19 infections to Jordan and to prevent local spread (Table S3 in the **Online Supplementary Document**). Control measures included public education, halt travel from endemic area, border checks, quarantine of suspected cases and isolation of confirmed cases, activation of a COVID-19 crises team, full closure of all borders (airport, land entry, and sea entry), prevention of gatherings and public events, preparation of COVID-19 isolation centers, medical staff, epidemiological investigation teams, and related resources, halt all educational institutions operations, and ended up by imposed a complete curfew and activation of the defense law and emergency state that is still active by 16th April 2020 [5,6] (Table S3 in the **Online Supplementary Document**).

## CLINICAL AND LABORATORY FEATURES OF COVID-19 IN JORDAN

### Clinical features

The following clinical features of COVID-19 are from analysis of 125 confirmed COVID-19 patients who were admitted to Prince Hamzah Hospital (PHH), the referral isolation center in Amman (Table S4 in the **Online Supplementary Document**). Among these COVID-19 patients admitted to PHH, the mean age was 26.6 ± 18.3 years, range (5 weeks to 73 years), with most patients 1-10 years (22.4%). Regarding gender 50.4% were males and 49.6% were females. Past medical history was documented in 36 patients (28.8%) (hypertension 10, diabetes 8, asthma 4, ischemic heart disease 4, systemic lupus erythematosus 2, and rheumatoid arthritis 2), past surgical history was documented in 47 patients (37.6%), allergic history was evident in 3 patients (2.4%), while current smoking was positive in 24 patients (19.2%) (Table S4 in the **Online Supplementary Document**).

The most common presenting symptoms were generalized malaise (49.6%), headache (47.2%), loss of smell (43.2%), diarrhea (40.8%), and loss of taste (40.8%). Only 32.8% reported fever, 36.8% reported dry cough, 21.6% reported wet cough, and 20% reported shortness of breath. The least frequently reported symptoms were chest pain (15.2%), palpitations (8.0%), and hemoptysis (0%) (Table S4 in the **Online Supplementary Document**). Severity of clinical condition indicated 13.6% were asymptomatic, 82.4% were mild to moderate, and only 4% were severe to critical.

### Laboratory features

Laboratory investigations of COVID-19 patients admitted to PHH (Table S5 in the **Online Supplementary Document**) showed low hemoglobin and hematocrit levels in 11.3%. Total white blood cells count was low in 8.5% and high in 5.7%. Differential count showed neutrophils percentage was low in 18.9%, lymphocytes percentage was low in 5.7% and high in 41.5%, basophils percentage was low in 51.9%, eosinophils percentage was low in 57.5%, monocytes percentage was high in 35.8%. Platelets count was low in 9.4%, with high PT, INR, and D-dimer levels in 2.8%, 4.7%, and 10.4% respectively. Inflammatory markers including CRP, ESR, and procalcitonin were elevated in 19.8%, 11.3%, and 3.8% respectively. Urea and creatinine were elevated in about 14%. AST, ALT, and LDH were elevated in 9.4%, 5.7% and 4.7% respectively (Table S5 in the **Online Supplementary Document**).

We report for the first time the epidemiological, clinical, and laboratory features of COIVD-19 patients in Jordan. Epidemiological analysis showed that the current COIVD-19 case-fatality rate in Jordan is low compared to global, regional, and Eastern Mediterranean countries, the recovery rate is high, and the number of total cases and death cases per million are low (**Table 1**) [1,2]. Most countries in the Gulf area including United Arab Emirates, Qatar, Bahrain and Kuwait have a relatively high number of cases (˃1000), however, the case-fatality rate is very low (<0.7%). Reporting accuracy is questionable for some Eastern Mediterranean countries. These numbers should be interpreted with caution as it evolves rapidly, there are discrepancies in case definitions, number of conducted tests, and reporting policies between countries. Furthermore, undiagnosed and asymptomatic cases, which are not included in the total number of cases, would have a significant effect on different rates, including case-fatality rate [1,2].

The epidemiological curve of active COVID-19 cases in Jordan showed a sharp increment for 2 weeks followed by flattening for about 1 week and ended by downward sloping in the last week. The total number of active cases remains under 250, and in the last week the number of recovered cases is higher than the number of new cases, while maintaining a very low death rate. This would indicate the effectiveness of applied control measures, decrease pressure on medical services, and ensure continuation of active epidemiological surveillance.

Regarding symptoms of COVID-19 patients in Jordan, the frequency of some symptoms was different from other studies. The frequency of fever and cough was low compared to multiple systemic review and meta-analysis studies which reported fever and cough as the most common symptoms [3,4,7,8]. On the contrary, rare symptoms including headache, nasal congestion, and diarrhea were reported at higher frequencies in our patients [3,4,7,8]. Of note, while anosmia and ageusia were not reported by COVID-19 patients in multiple systemic reviews and meta-analyses, a high frequency of our patients presented with these symptoms (˃40%) [3,4,7,8]. Gustatory and olfactory dysfunction were reported by three studies at about 5%, 19%, and 85% rates mainly in mild to moderate disease [9-11]. The frequencies of shortness of breath, generalized malaise, and myalgia were consistent with other studies [3,4,7,8]. Similarly, age and gender distribution, clinical condition of death cases, frequencies of chronic medical diseases, and smoking frequency in COVID-19 patients in Jordan were consistent with other studies [3,4,7,8].

In this study only 5.7% of COVID-19 patients had lymphopenia while 41.5% had lymphocytosis, this contrasts with most studies reporting lymphopenia association with COVID-19, and very few studies reporting lymphocytosis association [3,4,7,8]. Other laboratory data including inflammatory markers, coagulation and hemostasis markers, liver enzymes and kidney functions were elevated at frequencies less than most other studies [3,4,7,8]. This is possibly, due to a large number of asymptomatic and mild diseases reported in this study.

## CONCLUSIONS

The current total number of confirmed COVID-19 cases in Jordan, active cases, and death cases are low compared to global and regional rates. The recovery rate of COVID-19 patients is high, while the case-fatality rate is low. A higher number of recovered cases compared to new cases leads to the flattening of the COVID-19 epi-curve in Jordan reflecting effectiveness of control measures. Rare symptoms including anosmia and ageusia, headache, nasal congestion, and diarrhea were common presentations in COVID-19 patients in this study. Furthermore, atypical laboratory presentations were also noticed. Maintaining the flat curve while resuming public activities would be challenging.

## Additional material

Online Supplementary Document
