# Supplementary material for: Successful flattening of COVID-19 epidemiological curve in Jordan
Source: J Glob Health. 2020 Nov 8;10(2):020361. doi: 10.7189/jogh.10.020361 (PMC7688063; doi:10.7189/jogh.10.020361)
Supplement: Online Supplementary Document [file jogh-10-020361-s001.pdf]

Table S1. Daily Jordan COVID-19 total and new confirmed cases, total and new recovered cases, total and new death cases, and active cases (updated 16-4-2020)

Date	New cases	Total cases	Active cases	New recovered	Total recovered	New death	Total death
**2/3/2020**	1	1	1	0	0	0	0
**15/3/2020**	12	13	12	1	1	0	0
**16/3/2020**	16	29	28	0	1	0	0
**17/3/2020**	11	40	39	0	1	0	0
**18/3/2020**	12	56	55	0	1	0	0
**19/3/2020**	13	69	68	0	1	0	0
**20/3/2020**	15	84	83	0	1	0	0
**21/3/2020**	15	99	98	0	1	0	0
**22/3/2020**	13	112	101	0	1	0	0
**23/3/2020**	15	127	126	0	1	0	0
**24/3/2020**	26	153	152	0	1	0	0
**25/3/2020**	19	172	171	0	1	0	0
**26/3/2020**	40	212	210	1	2	0	0
**27/3/2020**	23	235	217	16	18	0	0
**28/3/2020**	11	246	227	0	18	1	1
**29/3/2020**	13	259	238	0	18	2	3
**30/3/2020**	9	268	237	8	26	2	5
**31/3/2020**	6	274	239	4	30	0	5
**1/4/2020**	4	278	237	6	36	0	5
**2/4/2020**	21	299	249	9	45	0	5
**3/4/2020**	11	310	247	13	58	0	5
**4/4/2020**	13	323	244	16	74	0	5
**5/4/2020**	22	345	230	36	110	0	5
**6/4/2020**	4	349	217	16	126	1	6
**7/4/2020**	4	353	209	12	138	0	6
**8/4/2020**	5	358	202	12	150	0	6
**9/4/2020**	14	372	204	11	161	1	7

**10/4/2020**	0	372	195	9	170	0	7
**11/4/2020**	9	381	197	7	177	0	7
**12/4/2020**	8	389	181	24	201	0	7
**13/4/2020**	2	391	169	14	215	0	7
**14/4/2020**	6	397	155	20	235	0	7
**15/4/2020**	4	401	144	15	250	0	7
**16/4/2020**	1	402	136	9	259	0	7

Table S2. Demographics of Jordan COVID-19 confirmed cases (n = 279, dated 31-3-2020)

	Variable	Number	Percentage (%)
**Gender**	Male	140	51
	Female	134	49
**Age (years)**	<5	8	2.91
	5-19	47	17.15
	20-59	178	64.96
	60-69	20	7.29
	>70	15	5.47
**Nationality**	Jordan	241	87.95
	Iraq	12	4.37
	France	6	2.18
	Lebanon	4	1.45
	United Kingdom	3	1.09
	Philippines	2	0.72
	Egypt	1	0.36
	Palestine	1	0.36
	Sri Lanka	1	0.36
	Uganda	1	0.36
	Spain	1	0.36
	Argentina	1	0.36
**City**	Amman	166	60.58
	Irbid	89	32.48

	Mafraq	3	1.09
	Petra	2	0.72
	Ajloun	2	0.72
	Salt	1	0.36
	Jarash	1	0.36
	Tourist	4	1.45
	Unknown	6	2.18
**Cases per week**	Week 9	1	0.36
	Week 10	0	0
	Week 11	83	30.29
	Week 12	151	55.1
	Week 13	39	14.23
**Source or cluster**	Irbid Wedding	47	17
	Hashmi	21	8
	Medical staff	17	6
	Hotels	11	4
**Hospital**	PHH	157	57.29
	KAUH	91	33.21
	Others	26	9.48

PHH - Prince Hamzah Hospital (Amman), KAUH - King Abdullah University Hospital (Irbid)

Table S3. Control measures applied to prevent the spread of COVID-19 in Jordan

Date	Measure
**27-2-2020**	• Jordan temporarily banns people entering from China, South Korea, and Iran
	• The kingdom starts screening everyone who enters at Jordanian border crossings and airports
**6-3-2020**	• Jordanian government makes an agreement with Facebook to launch an awareness campaign regarding COVID-19. Similar awareness campaigns are conducted by the Ministry of Health, media, private and governmental institutes.
**10-3-2020**	• Halt on travel to/from Lebanon, Syria, Israel and West bank
	• Closing of sea crossings with Egypt and reducing planes incoming from Egypt by 50%
	• Halting passenger movement through border crossings with Iraq
	• Jordanians returning from all the above will be allowed entry conditional to a self-imposed quarantine at home for 14 days
	• Cancellation of all international and local conferences
**11-3-2020**	• Preventing non-Jordanians from entering the Kingdom from the following countries: China, South Korea, Iran, Italy
	• Banning vacations for expatriates working in Jordan

	• Halting new work permits for workers from abroad
	• Banning vacations for foreign students studying in Jordan wishing to travel to countries where the disease has spread
	• Banning school trips to foreign countries
	• Suspending of public servant's travel to foreign countries
	• Suspending all tourist travel between Jordan and Palestine/Israel
	• Directing citizens arriving from countries with large numbers of COVID-19 cases (such as Iraq, Egypt, France, Spain, Germany) to self-quarantine at home for two weeks and alerting health authorities in case of any signs/symptoms of infection
**15-3-2020**	• Halting all educational institutions operations and start distance learning
	• Halting all public events and gatherings
	• Stressing on citizens to remain at home as much as possible and leave only if necessary
	• Halting prayer in all the Kingdom's mosques and churches
	• Halting of hospital and prison visits
	• Closing of all historic tourist sites
	• Halting all sports events, closing of all cinemas, swimming facilities, sports clubs and youth centers
	• Banning argeeleh (hookah) and smoking in coffee shops and restaurants
	• Activation of COVID-19 crisis team to work around the clock
**16-3-2020**	• Prevention of arrivals and Jordanians travel to/from France, Spain and Germany.
**17-3-2020**	• Full closer of all Jordanian Borders (airport, land entry, and sea entry) and compulsory quarantine of travelers in 5-star hotels (n= 5050 in 34 hotels) for 2 weeks under direct medical supervision
	• Halting work at all institutions and official departments, disable the private sector, stop the printing of paper newspapers, suspend the work of mass transportation, close all malls and commercial centers
	• Prevent the gathering of more than 10 people, and prevent movement between the governorates
	• Implementation of the National Defense Law
**19-3-2020**	• The Jordanian government declared a state of emergency
**21-3-2020**	• The Jordanian government imposed a curfew

Table S4. Demographic and clinical data of COVID-19 patients admitted to Prince Hamzah Hospital (n=125)

**Category**	**Variable**		**Number**	**Percentage (%)**
**Demographics**	Age	< 1	4	3.2
		1-10	28	22.4
		11-20	21	16.8
		21-30	21	16.8
		31-40	21	16.8
		41-50	15	12.0
		51-60	10	8.0
		61-70	4	3.2

		>70	1	0.8
	Gender	Male	63	50.4
		Female	62	49.6
**Symptoms**	Asymptomatic		17	13.6
	Generalized Malaise		62	49.6
	Headache		59	47.2
	Loss of Smell		54	43.2
	Diarrhea		51	40.8
	Loss of Taste		51	40.8
	Chills/Rigors		49	39.2
	Myalgia		48	38.4
	Nasal Congestion		48	38.4
	Dry Cough		46	36.8
	Fever		41	32.8
	Rhinorrhea		33	26.0
	Sweating		29	23.2
	Wet Cough		27	21.6
	Shortness of Breath		25	20.0
	Abdominal Pain		25	20.0
	Chest Pain		19	15.2
	Palpitations		10	8.0
	Hemoptysis		0	0.0
	Others		16	12.8
**Past Medical History**	Yes		36	28.8
	No		89	71.2
**Past Surgical History**	Yes		47	37.6
	No		78	62.4
**Allergic History**	Yes		3	2.4
	No		122	97.6
**Smoking**	Past smoker		5	4.0

	Current smoker	24	19.2
	None smoker	96	76.8

Table S5. Laboratory data of COVID-19 patients admitted to Prince Hamzah Hospital (n=106)

Investigations	Variable	Number	%
**Hb**	Low <12 g/dL	12	11.3
	Normal 12-16 g/dL	85	80.2
	High >16 g/dL	9	8.5
**HCT**	Low <35%	12	11.3
	Normal 35-47 %	87	82.1
	High >47%	7	6.6
**WBC**	Low <4000 /µL	9	8.5
	Normal 4000-11000 /µL	91	85.8
	High >11000 /µL	6	5.7
**Neutrophil %**	Low <40%	20	18.9
	Normal 40-80%	84	79.2
	High >80%	2	1.9
**Lymphocyte %**	Low <20%	6	5.7
	Normal 20-40%	56	52.8
	High >40%	44	41.5
**Basophil %**	Low <0.5%	55	51.9
	Normal 0.5-1%	47	44.3
	High >1%	4	3.8
**Monocyte %**	Low <2%	1	0.9
	Normal 2-10%	67	63.2
	High >10%	38	35.8
**Eosinophil %**	Low <1%	61	57.5
	Normal 1-6%	41	38.7
	High >6%	3	2.8
	ND	1	0.9
**Platelets count**	Low <150 000 /µL	10	9.4

	Normal 150 000-450 000 ⁄µL	93	87.7
	High >450 000 ⁄µL	2	1.9
	ND	1	0.9
**CRP**	Normal 0-5.0 mg/L	61	57.5
	High >5.0 mg/L	21	19.8
	ND	24	22.6
**Procalcitonin**	Low <0.10 ng/mL	1	0.9
	Normal 0.10 – 0.49 ng/mL	13	12.3
	High >0.49 ng/mL	4	3.8
	ND	88	83.0
**ESR**	Normal 0-15 mm/hr	19	17.9
	High >20 mm/hr	12	11.3
	ND	75	70.8
**PT**	Low <12 sec	5	4.7
	Normal 12-16 sec	47	44.3
	High >16 sec	3	2.8
	ND	51	48.1
**INR**	Low <0.85	0	0.0
	Normal 0.85-1.15	44	41.5
	High >1.15	5	4.7
	ND	57	53.8
**Urea**	Low < 2.86 mmol/L	8	7.5
	Normal 2.86 – 8.2 mmol/L	79	74.5
	High > 8.2 mmol/L	14	13.2
	ND	5	4.7
**Creatinine**	Low < 59 mmol/L	45	42.5
	Normal 59 – 104 mmol/L	41	38.7
	High > 104 mmol/L	15	14.2
	ND	5	4.7
**Sodium**	Low < 135 mmol/L	4	3.8

	Normal 135 – 152 mmol/L	96	90.6
	High > 152 mmol/L	0	0.0
	ND	6	5.7
**Potassium**	Low < 3.5 mmol/L	5	4.7
	Normal 3.5 – 5.3 mmol/L	82	77.4
	High > 5.3 mmol/L	4	3.8
	ND	15	14.2
**AST**	Normal ≤38 U/L	72	67.9
	High > 38 U/L	10	9.4
	ND	24	22.6
**ALT**	Normal ≤41 U/L	69	65.1
	High > 41 U/L	6	5.7
	ND	31	29.2
**LDH**	Low < 125 U/L	2	1.9
	Normal 125-378 U/L	17	16.0
	High > 378 U/L	5	4.7
	ND	82	77.4
**Alkaline Phosphatase**	Low < 40 U/L	6	5.7
	Normal 40-150 U/L	13	12.3
	High > 150 U/L	3	2.8
	ND	84	79.2
**D-Dimer**	Normal < 0.5 μg/mL	11	10.4
	High > 0.5 μg/mL	11	10.4
	ND	84	79.2

Hb - Hemoglobin, HCT - hematocrit, WBC - white blood cells, CRP - C-reactive protein, ESR - erythrocyte sedimentation rate, PT - prothrombin time, INR - The international normalized ratio, AST - aspartate transaminase, ALT - alanine transaminase, LDH - lactate dehydrogenase
